# Chest pain syndromes are associated with high rates of recidivism and costs in young United States Veterans

**DOI:** 10.1186/s12875-015-0287-9

**Published:** 2015-07-23

**Authors:** Basmah Safdar, James Dziura, Harini Bathulapalli, Douglas L. Leslie, Melissa Skanderson, Cynthia Brandt, Sally G. Haskell

**Affiliations:** Department of Emergency Medicine, 464 Congress Ave, New Haven, CT USA; VA Connecticut Healthcare System, 950 Campbell Ave, West Haven, CT USA; Yale Center for Analytical Sciences, 300 George Street, Suite 555, New Haven, CT USA; Penn State College of Medicine, A210, 600 Centerview Drive, Hershey, PA USA; Department of Internal Medicine, Yale School of Medicine, 333 Cedar St, New Haven, CT USA

**Keywords:** Chest pain, Recidivism, Costs

## Abstract

**Background:**

Recurrent chest pain is common in patients with and without coronary artery disease. The prevalence and burden of these symptoms on healthcare is unknown.

**Objectives:**

To compare chest pain return visits (recidivism) in patients with unexplained chest pain (UCP) against reference group of patients with coronary artery disease (CAD) and estimate the annual cost of recurrent chest pain.

**Methods:**

In a retrospective cohort study, a Veteran Affairs (VA) administrative and clinical database of Veterans who were deployed to or served in support of the wars in Iraq or Afghanistan was queried for first disease specific ICD-9 code to form two cohorts (UCP or CAD). Patients were followed between 09/2001-09/2010 for the first and cumulative return visits for UCP or cardiac pain (ACS or angina) to clinic, emergency department or admission; or for all-cause death. Time to return was analyzed using Cox regression and negative binomial models and adjusted for age, gender, race, marital status, and risk factors (hypertension, hyperlipidemia, diabetes, smoking and obesity). Direct total costs included inpatient, outpatient and fee basis (non-VA) costs.

**Results:**

Of 749,036 patients, 20,521 had UCP and 5303 had CAD. UCP patients were young and had a lower burden of risk factors than CAD cohort (p < .01). Yet, these patients were likely to return *earlier* with *any* chest pain (adjusted Hazard Ratio [aHR] = 1.76; 95 % CI 1.65-1.88); or unexplained chest pain than CAD patients (aHR: 1.89; 95 % CI 1.77-2.01). UCP patients were also likely to return *more frequently* for any chest pain (aRate Ratio = 1.54; 95 % CI 1.43-1.64) or UCP than CAD patients (aRR =2.63; 95 % CI 2.43-2.87). Per 100 patients, the 1-year cumulative returns were 37 visits for reference group and 45 visits for UCP cohort. The annual costs for chest pain averaged $69,009 for CAD and $57,336 for UCP patients (log geometric mean ratio=1.25; 95 % CI 1.18-1.32).

**Conclusion:**

Chest pain recidivism is common and costly even in patients without known CAD. We need evidence-based guidelines for these patients to minimize returns.

**Electronic supplementary material:**

The online version of this article (doi:10.1186/s12875-015-0287-9) contains supplementary material, which is available to authorized users.

## Background

Chest pain is one of the most common conditions seen in the primary care setting. In fact, 20-40 % of the population experiences some chest pain over their lifetime. In the United States, over eight million patients with chest pain are evaluated in the outpatient setting each year, posing a significant health burden [1]. Primary care physicians across the world face the challenge of distinguishing life threatening cardiac causes of chest pain from non-cardiac etiologies and of doing this in a timely and efficient fashion. The standard of care for chest pain patients is to first rule out acute coronary syndrome (ACS) [2, 3]. However, musculoskeletal causes of chest pain are the most common cause in primary care followed by gastrointestinal, pulmonary, psychological (depression or PTSD) or microvascular disease–all common in young patients [4–7]. Several diagnostic algorithms have been proposed to help the clinician reach a diagnosis in these cases [8]. And yet, a good proportion of cases remain ‘non-specific’ or ‘unexplained chest pain’ (UCP). This is evident from an upward trend in unexplained chest pain admissions nationwide despite declining ACS trends [9, 10]. In contrast to ACS patients, UCP is more common in young patients and in women corroborating their low-risk pre-test probability for CAD [9, 11, 12]. Deciding the appropriate level of response for such patients is both difficult task and variable, often leading to both over- and under-triage in primary care [13]. While the annual cost burden of heart disease has been estimated at $312 billion, similar costs of unexplained chest pain that represent majority of primary care visits remain unknown [14, 15].

We evaluated these questions in the Veteran Affairs (VA) health care system using the Women Veteran Cohort Study (WVCS), with its detailed electronic health records providing longitudinal data of service utilization and costs of Veterans of Operation Enduring Freedom (OEF), Operation Iraqi Freedom (OIF) and Operation New Dawn (OND) [16]. Prior reports show that chest pain is common among Veterans [17]. We chose this cohort because it primarily included young patients allowing us to estimate the burden of UCP in low-risk patients, and the single insurance with a well-integrated electronic medical records system allowed us longitudinal follow-ups. VA facilities located throughout the US allow Veterans to continue VA care despite relocation, thus allowing a prospective evaluation of the full burden and cost of unexplained chest pain. The main aim of our study was to document the burden of chest pain recidivism in young patients without CAD. Our hypothesis was the chest pain recidivism would be less in the low-risk UCP group compared to a reference group of patients with known CAD. The secondary outcome was calculating the annual costs associated with unexplained chest pain.

## Methods

### Study population and data sources

The Defense Manpower Data Center provided the VA OEF/OIF/OND roster for personnel discharged from the US military from 09/12/2001 to 09/30/2010 and enrolled for VA care. Data on their services and costs were linked to the VA administrative and clinical encounters to the VA National Patient Care Database, Decision Support Systems (DSS), National Data extracts and the VA Corporate Data Warehouse. Detailed information on services paid for by VA but provided by non-VA facilities or contract providers, including inpatient, outpatient and pharmacy care was obtained from the DSS Fee Basis files. The Human Investigation Committees at West Haven VA Medical Center and Yale University School of Medicine approved this study.

### Inclusion criteria and variable definitions

For diagnostic data, the International Classification of Disease, 9^th^ Revision (ICD-9) was used and medical conditions were included if the specific code was noted at least once for an inpatient stay, observation or ED stay or at least twice for an outpatient visit. This methodology for the use of ICD-9 codes in VA database has been validated elsewhere [18].

For co-morbidities, we used previously validated diagnostic algorithms for diabetes, hypertension, dyslipidemia, obesity, and CAD [19]. We defined dyslipidemia if patient had ICD-9 code for dyslipidemia or was on statins for a year. We included active smokers [20]. Body mass index was calculated using weight and height averaged over a 1-year period.

Study cohort was defined based on the *first* diagnosis after entry into the VA system (end of last deployment). Those with an encounter listed as ICD-9 code ‘786.50’ entered the unexplained chest pain cohort and those with CAD codes (see Additional file 1) formed known CAD cohort. There were 151 (0.007 %) patients in the first cohort that converted to CAD during follow up and we included only their follow-up to CAD conversion in the analysis.

### Outcomes and follow-up

Outcomes included any return visit to the VA or non-VA (fee basis) for either unexplained chest pain or cardiac chest pain (myocardial infarction, stable or unstable angina), or death. Patients were followed until study outcome or exit from the cohort during a ten-year study period. ICD-9 codes included were chest pain (786.50), CAD (414.x and 429.x), acute myocardial infarction (410.x), unstable angina (411.x) or angina (413.x) as primary or secondary diagnosis. Time to return visit as well as the cumulative number of chest pain visits were recorded.

Total health care costs were computed by summing all costs across inpatient, outpatient and pharmacy services after identification of cohort. Costs included both costs of services delivered in VA facilities as well as costs of services delivered by non-VA care(fee-basis) providers. Finally each of these measures were linked to the cardiac ICD-9 codes outlined above to determine the cost of care for both groups of patients.

### Analysis

Continuous variables were expressed as mean +/− standard deviation and compared by unpaired Student’s *t* test. Nominal or dichotomous variables were expressed as proportions and compared by chi-square test. Kaplan Meier curves were estimated to show those without a return visit by cohort and gender for (a) *any* chest pain (i.e., unexplained chest pain or cardiac chest pain) and b) unexplained chest pain only. Time to return visit and incident rate for death was compared using Cox regression with adjustment for age, gender, race, marital status, education, hypertension, dyslipidemia, smoking, diabetes, obesity (defined as BMI ≥30), cohort and interaction terms with gender.

Cumulative incident return rates were calculated per patient from the point of entry to last follow up. Crude incident rates were calculated by dividing the total number of return visits for chest pain by the total number of patients in each cohort for the duration of observation for each patient. All eligible index visits for chest pain were used as the unit of analysis. This way we captured multiple visits for the same patients. All rates were expressed as the total per 10000 visits. Rate ratios and confidence level for all rates were calculated using a generalized linear model with a negative binomial distribution and an offset of the log years of follow-up given the variable observation period for each patient. Separate adjusted models were constructed using the socio-demographic and cardiac risk factors listed above. The mean number of return visits per subject was calculated using a nonparametric estimator [21].

Total costs were compared across the two cohorts using geometric means and Mann-U-Whitney tests because of extreme right skewness. A generalized linear model with a log link function was developed to identify and adjust for differences in demographic, cardiac risk factors and year of entry (to account for inflation). Because pharmacy costs are not coded by diagnosis, all prescription use was compared for the two cohorts.

All analysis was performed using SAS v.9.0. P-values of <0.05 were considered statistically significant.

## Results

The study cohort followed 749,036 patients prospectively over ten years of which 2592 (2.9 %) females and 18,010 (2.7 %) males reported chest pain. After entering the VA system, 20,521 patients entered the unexplained chest pain (UCP) cohort and 5303 patients constituted the CAD (reference) cohort based on the first diagnosis and were analyzed. As expected, our cohort was young with median age 34 years (IQR 24, 42),11 % female and 66 % white. Overall cardiac risk factor profile was as follows: 39 % had hypertension, 12 % were diabetic, 45 % had dyslipidemia, 38 % smoked and 31 % were obese. Known CAD patients were more likely to be older, male, white, married and had a higher rate of co-morbidities (see Table 1).

**Table 1:** Baseline demographic characteristics of patients by gender and cohort

Characteristic	Coronary Artery Disease			Unexplained Chest Pain			Difference Between Cohorts
	(*n* = 5303)			(*n* = 20521)			
	21 %			79 %			
	Total	Female	Male	Total	Female	Male	P value
	CAD	6 %	94 %	UCP	13 %	87 %	
Median Age in years (IQR)	42 (34,49)	38 (26,44)	42 (34,49)	31 (23,40)	29 (23,39)	31 (23,40)	<.001
Race							
White	58 %	41 %	59 %	55 %	42 %	57 %	<.001
Black	19 %	38 %	18 %	21 %	36 %	19 %	
Hispanic	9 %	6 %	9 %	12 %	9 %	13 %	
Others	14 %	15 %	14 %	12 %	13 %	11 %	
Married	69 %	41 %	71 %	51 %	35 %	54 %	<.001
Educated (High School or higher)	99 %	99 %	99 %	99 %	99 %	99 %	0.02*
Cardiac Risk Factors							
Hypertension	50 %	31 %	51 %	29 %	21 %	30 %	<.001
Diabetes	14 %	7 %	14 %	5 %	4 %	6 %	<.001
Hyperlipidemia	57 %	34 %	58 %	33 %	22 %	35 %	<.001
Smoking	37 %	29 %	38 %	39 %	29 %	40 %	0.01
Obesity	33 %	30 %	33 %	28 %	25 %	28 %	<.001

Note: Table percentage are column percentage; *percentage are rounded off to the nearest decimal; the *p* is significant at > .05 level using Chi-sq Test

### Primary outcome

Patients were followed over an average of 2.02 years for the following:

### a) First return visit for chest pain

**Fig. 1:**
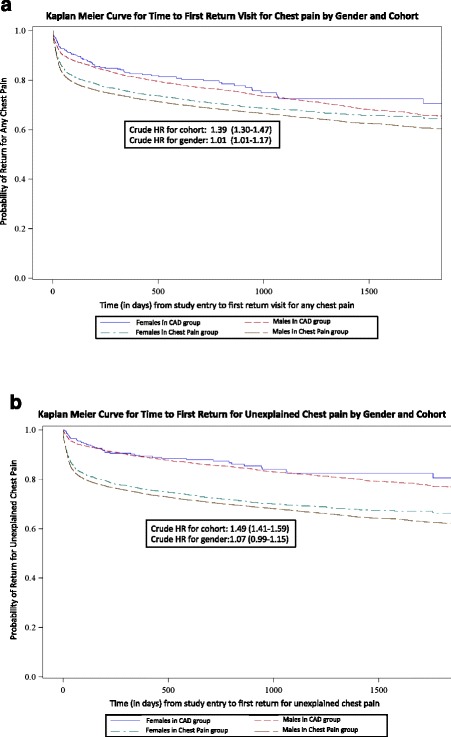
Kaplan-Meier Curves for Time to First return for Chest Pain By Gender and Cohort

The 1-month and 1-year probability of a return visit for *any chest pain* was 14.2 % and 26.6 % respectively in the unexplained chest pain cohort versus 8 % and 18.4 % in the CAD cohort (Crude HR = 1.39, 95 % CI 1.31-1.47) (Fig. 1a). Adjusted Cox regression showed UCP patients were 76 % more likely to return earlier with chest pain as compared to CAD patients (Adjusted HR = 1.76; 95 % CI 1.65-1.88).

The 1-month and 1-year probability of a return visit for *unexplained chest pain* alone was higher (13.4 % and 25.3 %) in the UCP cohort than the reference group (4 % and 10.8 %) (Crude HR = 1.49; 95 % CI 1.41-1.59) (Fig. 1b). After adjustment, UCP patients were 89 % more likely to return earlier for unexplained chest pain as compared to CAD patients (Adjusted HR: 1.89; 95 % CI 1.77-2.01).

### b) Cumulative returns for chest pain

Negative binomial regression comparing cumulative return visits for chest pain for the duration of follow up revealed that patients with UCP were 58 % more likely to return for any chest pain (Rate Ratio = 1.58; 95 % CI 1.47-1.71) and 2.6 times more likely to return with unexplained chest pain than the reference group (Rate Ratio = 2.63; 95 % CI 2.43-2.87) (Table 2). Two hundred and nine (0.01 %) patients died in UCP group compared to 106 (0.02 %) CAD patients (HR = 0.61; 95 % CI 0.45-0.83).

**Table 2:** Incidence rates for return visits by cohort

	Incidence Rate (per 10,000 person-years)		*Unadjusted Rate Ratio/Hazard Ratio (with 95 % confidence intervals)*	*P value*	*Adjusted Rate Ratio/Hazard Ratio* ^*a*^(with 95 % confidence intervals)	*P value*
Cohort	Unexplained CP	Known CAD				
Any Return for CP	8.2	7.1	1.25 (1.16-1.35)^b^	<0.0001^*^	1.54 (1.43-1.66)^b^	<0.0001^*^
Unexplained CP Return	7.5	3.9	2..23 (2.05-2.40)^b^	<0.0001^*^	2.63 (2.43-2.87)^b^	<0.0001^*^
Death	0.1	0.2	0.51 (0.39-0.68)^b,c^	0.0001^*^	0.61 (0.45-0.83)^b,c^	0.0018^*^

Notes: Rates area expressed in 10,000 person years; table percentages are column percentages; *the *p* is significant at > .05 level

^a^adjusted for age in decades, white race, higher education, marital status, hypertension, diabetes, hyperlipidemia, smoking and obesity; there was no interaction found between gender and cohort

^b^ratio of rates of return visits in Unexplained chest pain cohort/CAD cohort

^c^Cox regression model used to calculate hazard rates

**Fig. 2:**
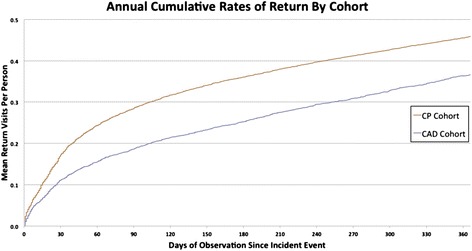
Annual Cumulative Rates of Return for Unexplained Chest Pain by Cohort

Figure 2 shows the cumulative return visits for any chest pain by cohort. The 30-day mean return visits were 11 visits per 100 CAD patients and 17 visits per 100 UCP patients. The rates were consistently 12-15 % higher in the UCP cohort compared to the reference group at 3 months, 6 months and at 1 year from the index visit (Fig. 2).

The adjusted time to first return or cumulative rates of return visits did not differ by gender (Figs. 1 and 2).

### Secondary outcome

#### Cost comparison

**Fig. 3:**
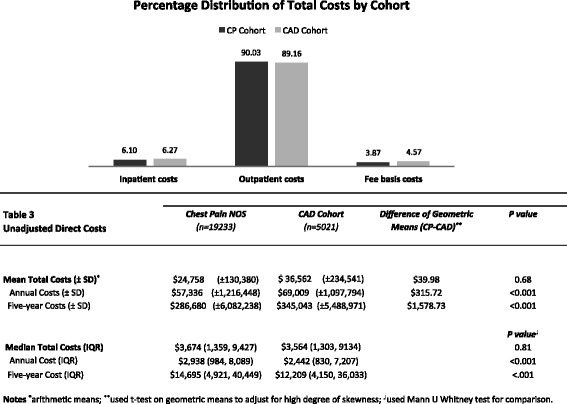
Unadjusted Direct Costs of Unexplained Chest Pain

Figure 3 shows mean and median costs for UCP patients that were comparable to those of patients in the CAD cohort. Mean costs were averaged across all follow up years including those with zero costs. The mean per patient total cost was $11,804 higher in the CAD cohort as compared to the UCP cohort. Given highly skewed data, we compared the cohorts using geometric means as well as winsorized means at 5 % and 10 % cut off values and did not find a difference (results not shown).

After adjustment for socio-demographics and cardiac risk factors using a generalized linear model with a log link function, CAD patients incurred 25 % higher costs than patients with unexplained chest pain (Log geometric mean ratio=1.25; 95 % CI 1.18-1.32). Costs were heavily skewed, as the median costs as well as distribution of costs were similar between both groups (Fig. 3). Figure 3 shows the percent distribution of total costs across care setting for the two cohorts. Costs for procedures were 16.89 % of the total costs for UCP cohort and 16.18 % for the CAD cohort, while total pharmacy costs were 10.13 % of the total costs for the UCP cohort and 13.89 % for the CAD cohort.

## Discussion

To our knowledge, this is the first US study that has quantified the recidivism and cost burden of unexplained chest pain in young patients. In a geographically representative national database, we found that young Veterans without coronary disease had recurrent chest pain earlier and 1.5 times more frequently than the reference group with known CAD. Studies from the UK and Norway have shown similar high burden of unexplained chest pain [13, 22]. We also report the cost burden of unexplained chest pain in the VA system. Although the adjusted average cost of chest pain visits was 25 % higher with CAD, median costs and distribution of inpatient, outpatient and non-VA care (fee basis) costs of chest pain were comparable in both cohorts. With the Affordable Care Act of 2010 linking readmissions to hospital reimbursement and value-based purchasing, we believe this information is of value to hospitals and clinicians.

Our study adds several interesting findings to the existing literature. First, we found prevalence of unexplained chest pain to be high in young Veterans without coronary artery disease. This is consistent with reports from civilian population that show chest pain syndromes are more common in young patients [4, 22–25].

Second, we found chest pain recidivism to be high in young Veterans. The 30-day return rate was twice in our cohort compared to civilian reports [26]. Chest wall syndromes are often associated with high recurrence rates [27]. Patients with unexplained chest pain often view their condition as significantly less controllable and less understandable than those with pain of cardiac origin [28]. The CAD conversion rate was very low in our cohort suggesting non-cardiac cause of recurrent chest pain. Our prior work documented high rates of depression (30 %) and PTSD (17 %) as well as musculoskeletal conditions in the WVCS cohort that could contribute towards recurrent non-cardiac chest pain [16]. Depression and anxiety has been linked with increased rates of recurrent chest pain, either through increased somatization or by reducing release of nitric oxide and potential decrease in endothelial reactivity [29, 30]. It is also possible that some of these patients returned due to uncertainty of diagnosis. Prior reports have shown that increased awareness of the possibly dangerous ramifications of chest pain may drive patients back to the hospital for recurrent symptoms despite an initial cardiac work up [31]. In this study, we have quantified the rates of recidivism. We did not collect data on diagnostic work-ups on these patients and it is possible that due to the lower pretest probability these young Veterans did not get a comprehensive ‘rule out ACS’ evaluation prompting early returns. However such approach would not entirely explain the high annual costs incurred by this cohort or the high cumulative rates of return visits.

Finally, the cost implications of recurrent chest pain are important. Prior reports demonstrate escalating costs of unexplained chest pain admissions over the past decade [9]. In this study, we report additional outpatient costs of UCP in comparison with CAD costs in the VA system. We found the cost of recurrent chest pain to be high despite low pre-test probability of CAD in young Veterans. Annual inpatient cost averaged $3498 per patient for chest pain and $4327 for CAD. This was similar to the national average cost of unexplained chest pain ($4014) as paid by Medicare [32]. The primary cost driver in the VA was outpatient visits (clinics, emergency departments and urgent care), where 80 % of chest pain patients are ruled out for ischemia. We recognize the financing mechanisms that are unique to the VA, however these are important hypothesis generating results that should be investigated in the civilian population. If corroborated in the six million annual civilian chest pain ED visits, at an annual average total cost of $57,336 for unexplained chest pain per patient, our study projects an annual national health burden of up to $344 billion due to recurrent chest pain. This would be in addition to the $312 billion cost of CAD based on prior reports of admission costs [33].

For practicing clinicians, we feel that the large population base investigated in our study, together with evidence from other studies, highlights the need to recognize unexplained chest pain as a heterogeneous syndrome and the relative importance of continuing work up for a definitive diagnosis. Our present system evaluates acute chest pain primarily through an ‘ACS lens’–understandable given that missed myocardial infarction is associated with high mortality [34]. However the CAD conversion rates in our young cohort remained low (0.007 % UCP patients converted to CAD) corroborating their low-risk for CAD. This coupled with low mortality in this group underscores the need to look beyond tier one outcome (survival) in young patients with recurrent chest pain. Some authors have proposed diagnostic algorithms to facilitate work ups and even cost saving strategies such as initiation of high dose gastric acid suppressive therapy after ruling out cardiac causes [8, 35]. We need more research to better quantify these causes and develop evidence-based guidelines for managing persistent chest pain that are value-based, patient centered and integrated towards a ‘rule in’ strategy [36]. Documenting the prevalence and cost of this condition is the first step in this direction.

### Limitations

Our study has several limitations. First, we used an administrative database that relied on ICD-9 codes and did not capture the granularity of diagnostic work ups. The findings are therefore subject to the accuracy and subjectivity of ICD code documentation as well as presumed adequacy of patient work ups. Further work is needed to determine the definitive diagnoses eventually given to these patients. Second, the costs cannot be generalized to all Veterans because the data were limited to those seeking VA care. However our cohort was young (75 % less than 50 years) and unlikely to have alternate insurance such as Medicare. Our results may then actually underestimate the rates of return visits. Third, it is possible that some patients were redeployed during the follow up period, however symptomatic patients with chest pain are unlikely to be deployed. Fourth, it is possible that coding errors occurred. However our findings from previous studies show good reliability [18]. Fourth, this VA cohort is young and unique that could affect the generalizability of our findings. However UCP is also common in younger cohorts in patients largely free of CAD as reported by Fagring et al. from a population-based study [12]. It is also interesting to see that despite lower pretest probability of CAD, this young cohort incurred high costs of care. Fifth, we only used all cause-mortality in our outcomes and did not compare the reason of death. However, given the low mortality rates in this cohort, it may not influence the interpretation of our findings. Finally our study was based on physician diagnosis rather than patient-reports, and therefore likely represents and underestimate of the burden of disease [7].

## Conclusion

Unexplained chest pain is common and a frequent cause of recidivism in young Veterans without known CAD. With a projected annual cost of over $300 billion, unexplained chest pain represents an unidentified source of major cost to the health system and warrants further investigation.

### Presentations

Abstract from this study has been accepted for presentation at the American Heart Association Scientific Session 2013 to be held in Dallas, TX.

## Additional file

Additional file 1: **ICD-9 Codes for CAD.**

## Abbreviations

CAD: Coronary artery disease
UCP: Unexplained chest pain
ACS: Acute coronary syndrome
VA: Veteran Affairs
aHR: Adjusted hazard ratio
aRR: Adjusted Rate ratio
PTSD: Post traumatic stress disorder
WVCS: Women Veteran Cohort Study
OEF: Operation Enduring Freedom
OIF: Operation Iraqi Freedom
OND: Operation New Dawn
DSS: Decision support system
ICD-9: International Classification of Diseases -9^th^ edition
IQR: Inter-quartile range
