# Supplementary material for: Chest pain syndromes are associated with high rates of recidivism and costs in young United States Veterans
Source: BMC Fam Pract. 2015 Jul 23;16:88. doi: 10.1186/s12875-015-0287-9 (PMC4511555; doi:10.1186/s12875-015-0287-9)
Supplement: Supplementary file 1 — ICD-9 Codes for CAD. [file 12875_2015_287_MOESM1_ESM.pdf]

Appendix

ICD-9 Codes for CAD

**410**	**Acute MI**
410.0	Of anterolateral wall
410.00	Of Anterolateral wall – episode of care unspec
410.01	Of ant wall – initial ep of care
410.02	Of ant wall – subsequent episode
410.1	Of other anterior wall
410.10	Episode of care unspecified
410.11	Initial episode of care
410.12	Subsequent episode of care
410.2	Of inferolateral wall
410.20	Episode of care unspecified
410.21	Initial episode of care
410.22	Subsequent episode of care

410.3	Of inferoposterior wall
410.30	Episode of care unspecified
410.31	Initial episode of care
410.32	Subsequent episode of care
410.4	Of other inferior wall
410.40	Episode of care unspecified
410.41	Initial episode of care
410.42	Subsequent episode of care
410.5	Of other lateral wall
410.50	Episode of care unspecified
410.51	Initial episode of care
410.52	Subsequent episode of care
410.6	True posterior wall infarction

410.60	Episode of care unspecified
410.61	Initial episode of care
410.62	Subsequent episode of care
410.7	Subendocardial infarction
410.70	Episode of care unspecified
410.71	Initial episode of care
410.72	Subsequent episode of care
410.8	Of other specified sites
410.80	Episode of care unspecified
410.81	Initial episode of care
410.82	Subsequent episode of care
410.9	Unspecified site
410.90	Episode of care unspecified
410.91	Initial episode of care

410.92	Subsequent episode of care
411	Acute and subacute forms of ischemic heart disease
411.0	Postmyocardial infaction syndrome
411.1	Intermediate coronary syndrome
411.8	Other acute and subacute forms of ischemic heart disease
411.81	Coronary occlusion without mi
411.89	Other
412	Old myocardial infaction
413	Angina pectoris
413.0	Angina decubitus
413.1	Prinzmetal angina
413.9	Other and unspecified angina pectoris

414	Other forms of chronic ischemic heart disease
414.0	Coronary atherosclerosis
414.00	Of unspecified type of vessel, native or graft

414.01	Of native coronary artery
414.8	Other specified forms of chronic ischemic heart disease
414.9	Chronic ischemic heart disease unspecified

429.7	Certain sequelae of myocardial infarction, not elsewhere classified
V45.81	Aortocoronary bypass status

V45.82	Percutaneous transluminal coronary angioplasty status
